# Genes and life-style factors in BELFAST nonagenarians: Nature, Nurture and Narrative

**DOI:** 10.1007/s10522-015-9567-y

**Published:** 2015-03-14

**Authors:** Jennifer Nicola M. Rea, Ashley Carvalho, Susan E. McNerlan, H. Denis Alexander, Irene Maeve Rea

**Affiliations:** 1 Research Department Primary Care and Population Health, University College London, London, UK; 2 School of Medicine, Dentistry and Biomedical Science, Queens University Belfast, Belfast, Northern Ireland, UK; 3 Regional Cytogenetics Laboratory, Belfast Health and Social Care Trust, Belfast, Northern Ireland, UK; 4 School of Biomedical Science, University of Ulster, Coleraine, Northern Ireland, UK

**Keywords:** BELFAST nonagenarians, Genes, Life-style, Nurture, Nature, Narrative

## Abstract

Understanding how to ‘*Age Longer and Age Well*’ is a priority for people personally, for populations globally and for government policy. Nonagenarians are the oldest members of our societies and survivors of their generation. Approximately 10 % of nonagenarians reach 90 years and beyond in good condition and seem to have a combination of both age-span and health-span. But what are the factors which help people reach their ninetieth birthday and beyond in good condition? Are they genetics, as in ‘*nature*’, or do they depend on ‘*nurture*’ and are related to environment, or are both factors inextricably intertwined within the concept of behavioural genetics? Nonagenarians have rich life experiences that can teach us much about ageing well; they are reservoirs of genetic, life-style and behavioural information which can help dissect out how to live not only longer but better. Personal family history and narrative are powerful tools that help to determine familial traits, beliefs and social behaviours and when used in parallel with new biotechnology methods inform and elaborate causality. Here we present themes and insights from personal narrative enquiry from nonagenarian participants from the Belfast Elderly Longitudinal Free-living Ageing STudy (BELFAST) about factors they consider important for good quality ageing and relate these insights to the emerging genetics and life-style evidence associated with healthy longevity.

## Introduction

Mixed method research, using both quantitative and qualitative aspects, has the potential to ask contrasting and distinctive questions about the interface between the scientific and social world, and to gain understanding about how the evidence from biological and life sciences can be acted out in the real world of individual beliefs and behaviours. Those differences—and relationships between the interdisciplinary fields of life sciences and those of social science, arts and humanities—help to bring the relevance of science into the socio-economic world of everyday life, because as a recent editorial in Nature argues ‘*science doesn’t advance far if we don’t understand the dynamics of behaviour, attitude and organisation*’ (Editor 2015).

Present day social science is increasingly recognising the validity of the nonagenarian insights which are telling us powerful messages about their ageing and are directly in line with present-day public health campaigns-encouraging populations to adopt better lifestyles and behaviours in order to make for a healthier life course.

Therefore in the BELFAST nonagenarian study, it was of interest to identify what range of behaviours, interventions and self-management strategies had supported and contributed to the BELFAST ‘elite’ nonagenarian’s combination of age-span and health-span, and which exemplified the Perl ‘escaper’ phenotype (Evert et al. 2003). Through narrative enquiry, we aimed to get a sense of the nonagenarian knowledge and engagement with their own good quality ageing, in terms of life-style and behaviours.

Here we discuss the main themes which the nonagenarian themselves identified as important pointers in their age-span and health-span, relate these to some present day evidence about genes and life style interactions related to longevity and discuss how the combined evidence supports everyone adopting better ageing strategies.

## Subjects and methods

In the BELFAST study, 90 year old subjects, who were ‘*very good*’ for their age, also called ‘*elite*’ and met the Senieur protocol (Lightart et al. 1984), were recruited through their General Practitioners, from the Greater Belfast area. Subjects willing to enrol, were community-living, mobile, and mentally competent (mini mental score examination > 26/30), (Folstein et al. 1975) and gave written consent. Briefly, subjects gave blood samples for DNA and other laboratory variables, responded to nutrition, life style and medical history questionnaires, blood pressure and anthropometric measurements (Rea et al. 2009) and provided self-directed narrative life-stories (Ganzevoort and Bouwer 2007) together with structured questions (Rea and Rea 2011; Rea Maeve 2013). Ethical permission for the Belfast Elderly Longitudinal Free-Living Ageing STudy (BELFAST) studies was given by Research Ethics Committee Northern Ireland (ORECNI), 08/NIR03/42 and by The Queens University Belfast.

## Nonagenarians narrative insights about their longevity

Using grounded theory and qualitative analysis (Glaser and Strauss 1967; Strauss and Corbin 1990), five key findings and insights were identified by nonagenarians themselves as important in their long lives and good quality health (Rea and Rea 2011; Rea Maeve 2013).

The five main themes were identified by BELFAST nonagenarians as important factors in their longevity included (1) genes (2) diet (3) good health (4) physical activity (5) social networks and resilience. Each will be discussed briefly in the context of some of the present day evidence relating to longevity.

## Genes and longevity

### Apolipoprotein E gene (ApoE)

Many nonagenarians considered that the genes within their extended families were important factors contributing to their long lives.

The ApoE gene is one of the most important and consistent genes identified with respect to both healthy longevity and age-related disorders. Age-related diseases such as Alzheimers, and vascular dementia (Corder et al. 1993; Licastro et al. 2007; Chuang et al. 2010) and cardiovascular risk (Eichner et al. 2002; Ilveskoski et al. 1999) have been regularly associated with the ApoE gene and particularly the ApoE4 allele which tracks with higher lipoprotein values (Bennet et al. 2007). In parallel studies ApoE has also been identified as the single most important gene associated with ‘healthy longevity’ in a host of individual studies including locally in BELFAST nonagenarians (Bennati et al. 2010; Rea et al. 2001), several meta-analyses and most recently in the European Genetics of Healthy Ageing (GeHA) nonagenarian sibling cohort where it is closely related to the TOMM 40 gene location (Beekman et al. 2013; Deelan et al. 2011; Deelen et al. 2014), which is mostly associated with the ApoE4 variant (Brooks-Wilson 2013). Early research by Schachter et al. (1994) had identified ApoE2 allele as being more frequent in French nonagenarians and this finding has been confirmed in other studies where not only was ApoE2 allele frequency accentuated but carriage of the ApoE4 allele was reduced in nonagenarian and centenarians cohorts (Frisoni et al. 2001; Kervinen et al. 1994; Cauley et al. 1993; Eggertsen et al. 1993; Corder et al. 1996). In keeping with this hypothesis, BELFAST nonagenarians, showed a fall in ApoE4 allele frequency reduced from 16 % in <65 year olds from the same geographical area, enlisted for the MONItoring of CArdiovascular (MONICA) project, down to 8 % in BELFAST nonagenarians. There was a reciprocal change in ApoE2 allele frequency rising to 12 % in nonagenarians compared to 8 % in <65 year old MONICA local subjects (Rea et al. 2001). One of the main explanations for these findings has been the association of ApoE4 carriage with a more risky lipoprotein profile (Bennet et al. 2007) which likely contributed to earlier mortality through vascular disease affecting the brain and cardiovascular system. Hence the frequency of the ApoE4 allele showed attrition in the oldest healthiest nonagenarian and centenarian cohorts.

Added to this disease risk profile of the ApoE4 allele, the GeHA study of nonagenarian siblings (Beekman et al. 2013), clearly replicated the known stratification of ApoE frequencies North–South in Europe with higher population frequencies of ApoE4 in Northern Europe compared to Southern countries. This finding also appeared to track with the increased susceptibility of cardiovascular disease risk. It is also known that ApoE4 frequency is much lower in some areas of the world (Corbo and Scacchi 1999) with a baseline frequency reported of 10 % at age 20 falling to 4 % in the >1990s in Han Chinese, suggesting a similar attrition with age for ApoE4 allele but from a lower baseline population frequency (Jian-Gang et al. 1998; Lu et al. 2014). Again there is the consideration that this lower ApoE population frequency may associate with a lower baseline frequency and risk of cardiovascular disease early mortality.

## Diet, genes and life-style

### ApoE and life-style factors

The allelic variants of ApoE are associated with increasing cholesterol and lipid profile in the range E4 > E3 > E2 so that E4 carriers have higher lipoprotein fractions and associated increased risk of cardiovascular disease, dementia and stroke, compared to E2 carriers (Bennet et al. 2007). BELFAST nonagenarians who carried the ApoE4 allele showed higher serum cholesterol values compared to those who carried the ApoE2 allele in keeping with the finding of increased vascular-related risk and attrition effect on the ApoE4 gene pool in 90 year old survivors. Changes in modern-day lifestyle have been argued to be important in the present-day and atherosclerotic-related risk for ApoE function in Western populations who have adopted higher fat diets and a less physical life-style. Raichlen and Alexander 2014 have argued and provide evidence that ApoE4, which is the ancestral variant, is negatively affected by smoking, high fat diet and a sedentary life-style but can be modulated by appropriate excise and diet regimes similar to those which were part of the life-style of our ancestors. Emphasising the same theme, Mattson (2012) argues that the fast food and the doorstep pizza delivery was not part of the life-style of our ancestors who had to spend much more physical energy in obtaining their daily energy needs (Mattson 2012).

One of the biggest conundrums is the association of the ApoE gene with both longevity and age-related disease (Perls 2002; Zhang et al. 2008). Does this suggest that the life style factors which BELFAST nonagenarians or the Perl ‘escaper’ phenotype (Evert et al. 2003) have adopted are instrumental in delaying age-related diseases and vascular risk (Hagberg et al. 2000; Masson et al. 2003; Ordovas 2008) and that through a combination of behavioural genetics and life-style factors, ‘elite’ nonagenarians intuitively chose the different life experiences, diets and lifestyles best suited to them (Gibney and Walsh 2013)?

### TCF7L2 gene and Mediterranean diet

The relationship between the TCF7L2 and the Mediterranean diet is one of the most recognised gene/diet interactions (Corella et al. 2013). Strict compliance with the Mediterranean diet can modulate the damaging effects of the T risk allele which is an important risk factor for development of diabetes.

Importantly, in the context of longevity, Garagnani et al. (2013) argued that centenarians could be used as ‘*super controls’* to assess the biological significance of genetic markers for age-related diseases, for Type 2 diabetes and the TCF7L2 genotypes. The group reported a marked reduced frequency of the diabetes-related risk T allele of TCF7L2 in their super centenarian controls, but an enrichment of the homozygous CC genotype, suggesting that the CC genotype could be a strong protective variant, at least in Italian centenarians likely to have been exposed to a Mediterranean diet.

## Good health and immunity

### Cytokine genes

Cytokine genes control cytokines, which in turn drive the immune response (McNerlan et al. 2009). Together they orchestrate and maintain the immune system thorough out life and seem likely to have an important role in good quality ageing and longevity. Cytokine polymorphisms have functional effects which determine serum cytokine responsiveness to danger signals, including infections, cancerous cells, toxins, diet and exercise (Ross et al. 2003; Rea et al. 2006). Considering the continual pressures from internal and external stressors, the immune system’s adaptability is being constantly shaped and re-shaped thorough out life. It seems likely that there are different cytokine gene polymorphisms and phenotypes, some of which contribute to the age-related disease phenotype and are perhaps associated with a strongly pro-inflammatory response, which could contribute to survival from childhood infections, but might drive immune activation with a predilection to age-related disease later in life (Rea et al. 2006), while conversely another cytokine phenotype could be associated with an accentuated anti-inflammatory profile, a more modulated immune response to mid life ‘*stressors’* and/or age-related disease and contribute to a cytokine profile, better shaped to facilitate longevity (Lio et al. 2002).

### IL-6 and Mediterranean diet

The IL-6 gene is called the *gerontologist cytokine* (Ershler 1993) and was considered a likely candidate in longevity and good quality ageing, because most age-related diseases are associated with increases in serum IL-6. Subjects homozygous for the risk allele (C) of the-174 G/C IL-6 polymorphism have higher levels of IL-6 than do G genotype carriers and a demonstrably increased risk from cardiovascular disease (Spoto et al. 2014). It was therefore argued that the G-allele-carriers might be more common in those who became nonagenarians or centenarians. In the local BELFAST nonagenarian cohort, the frequency of homozygous IL-6 G allele showed some attrition in very aged persons but was insufficiently powered (Rea et al. 2003). A subsequent European meta-analysis demonstrated a shift in IL-6-G-allele frequencies North/South but showed no significant association with longevity in the combined aged-cohorts (Di Bona et al. 2009). However a separate analysis of southern European centres showed an increased odds ratio for the G-allele and longevity (Di Bona et al. 2009). Here it was postulated that the IL-6 gene polymorphisms could be modulated by the Mediterranean diet as has been noted in cellular studies (Mena et al. 2009; Camargo et al. 2010) with the Mediterranean diet contributing to differences between Northern and Southern European incidence, and mortality from vascular disease (Fung et al. 2009; de Lorgeril and Salen 2006).

### Autoimmune disease and Mediterranean diet

Similarly there is increasing evidence that autoimmune diseases such as rheumatoid arthritis or lupus or ulcerative colitis may be modulated by compliance with a Mediterranean type diet (Skoldstam et al. 2003; McKellar et al. 2007) which appears to reduce the inflammatory profile of disease through mechanisms which are considered to be mediated through down-regulation of the NFkB pathway, which stands at the cross-roads of the cellular inflammatory cascade (Lawrence 2009).

### Diet, exercise and anti-oxidant status

In exercise-related stress there is evidence that anti-oxidant capacity is important in reducing post-exercise stress (Radak et al. 2008) and that a careful control of anti-oxidant flux through diet may help to reduce damaging post-exercise cytokine responses (Lamina et al. 2013). While a moderate amount of exercise is considered good (Cobley et al. 2014; Radak et al. 2005) endurance and ultra marathon type sports can contribute to long-term damage and immune activation which does not settle between episodes and the positive and beneficial effects of ‘hormesis’ are lost (Lushchak 2014).

### Genetics and NK cells

Natural Killer cell (NK) populations have been found to be increased with increasing age (Miyaji et al. 1997; Sansoni et al. 1993; McNerlan et al. 1998). NK cells, together with related NKT subsets, the killer cell immunoglobulin-like receptor (KIR) receptor-gene-complexes and associated cytokine profiles, are highly important in effective in patrolling, controlling and protecting our immune landscape thorough out life from viruses, cancerous and damaged cells of all kinds (Peralbo et al. 2007; Rea et al. 2013. Their roles and interactions are likely to be important in maintaining immune integrity in people who live successfully into their 1990s and beyond (Mocchegiani and Malavolta 2004) and fit the criteria of the Perl ‘escaper’ model of successful ageing (Evert et al. 2003). The KIR genes control the functions of NK cells through A or B haplotypes, with A having a more inhibitory role on NK function, compared to the more activating role of B the KIR haplogroup. BELFAST nonagenarians grouped by A and B KIR haplotype showed a predominantly B haplogroup inflammatory cytokines profile for B haplogroup carriers but increased numbers of NK cells for nonagenarians carrying the KIR A haplogroup (Rea et al. 2013; Maxwell et al. 2004). These differences in KIR haplotype effects may be important in explaining the longevity phenotype with the increased number of NK and NKT-related cells found in nonagenarians (McNerlan et al. 1998; Peralbo et al. 2007) or conversely with the pro-inflammatory background found with increasing age or inflamm-ageing (Franceschi et al. 2000).

### NK cells and diet

 NK cells do appear to have a relationship with nutrition and diet. Their cytolytic activity of NK receptors have shown to be upregulated by nutrition measures including Vitamin D and anthropometric markers (von Essen et al. 2010; Cantorna et al. 2012). Scientists have found that vitamin D is crucial to activating our immune defences and that without sufficient intake of the vitamin D the killer cells of the immune system are not able to react to fight off serious infections in the body (Al-Jaderi and Maghazachi 2013). In preliminary work, there is a small negative relationship between NK cell number and BMI in BELFAST nonagenarians (Rea et al. 2013) which replicates findings between BMI and anthropometric measurements identified previously in Italian aged cohorts (Mariani et al. 1999; Ravaglia et al. 2000). This finding could be important in relation to the *obesity paradox* which has described improved outcomes from life-threatening serious infections and major surgical procedures in elderly people with higher BMIs (Hogue et al. 2009; Kuperman et al. 2013).

## Physical activity

Many of the nonagenarian cohort reported continuing to be physically active thorough out their lives. The nonagenarians are no longer alone in their belief that one of the secrets of survivorship is working hard and maintaining physical activities. The Cambridge University study of 334,000 people found that even a modest amount of physical activity prolonged life (Ekelund et al. 2015), with similar findings in another large pooled cohort analysis (Moore et al. 2012). A prospective observational study involving almost half a million Taiwanese reported that being active for as little as 15 min a day can add as much as 3 years to the length of life (Wen et al. 2011) and a major study in USA has further shown that maintaining active exercise was an important contributor to good quality ageing for male physicians, who reached 90 years of age in good health (Yates et al. 2008). In a recent systematic review of physical activity and healthy ageing including cognitive function, there were clear outcome findings suggesting that late-life physical activity is beneficial for cognitive function in elderly people, with three studies reporting a dose–response relationship between physical activity and cognition (Carvalho et al. 2014). Although mechanisms remain to be fully identified, brain-derived neurotrophic factor (BDNF) is known to be heavily involved in the differentiation, extension, and survival of neurons in the hippocampus, cortex and cerebellum during brain development (Neeper et al. 1995; Vaynmana et al. 2006), and some animal models (Rhyu et al. 2010; Patten et al. 2013; Merkley et al. 2014) followed by clinical studies, demonstrate that BDNF level is associated with hippocampal volume and with aerobic exercise (Pang and Hannan 2013; Lojovich 2010; Muscari et al. 2010; Erickson et al. 2011).

These studies all add to the accumulating evidence that says that exercise is good for everyone (Ploughman 2008), irrespective of age and may also keep our brains in sharper function by stimulating and maintaining neurogenesis.

## Genetic and molecular mechanisms of resilience

### Social networks and resilience

Successful ageing is considered to be more than the “absence of disease and maintenance of high functioning” (Baltes and Baltes 1990), but involves the active engagement in everyday social activities.

The value of good social networks and family interactions has been previously described as important in good quality ageing (Evert et al. 2003), with the number of social interactions daily and inclusion in family networks making for good mental health and social well-being (Rowe and Kahn 1997).

Although much remains to be clarified, the mechanisms through which stress can affect mental and physical health and resilience are becoming clearer. Excessive glucocorticoids (GCs) released after early life stress exposure seem to cause long-lasting destabilisation of the stress hormone system which in turn increases risk for later psychiatric disorders. These findings follow on the early work in animal studies whereby poor maternal care was shown to cause dysregulation of the HPA axis in rodents with altered transcription of the GC receptor gene (Meaney 2001). Absence of early nurturing, through licking behaviour in rodent models, led to changes in the promoter of the GC receptor NR3C1 gene (Meaney and Szyf 2005), with similar changes demonstrated in people with bipolar disorders and later life stress which correlated with frequent general practice attendance (Furukawa et al. 1999; Glaser et al. 2006; Francis et al. 1999).

Around this theme, nonagenarians reported ‘*being happy*’, ‘*always cheerful*’, ‘*never melancholy*’ and having a contentment with a ‘*rich life*’ and family relationships ‘*thank God I have such good children’*. Social networks and supportive relationships are known to buffer the effects of stressful life events such as bereavement, deteriorating health and loss of autonomy (Northern Arizona University 1998) and to mitigate negative feelings and emotions (Rentoul 1997).

## Summary comments

The themes emerging from collected life stories collected of ‘elite’ nonagenarians provide some insights into why nonagenarians believe they have lived so long and well. Their narratives and answers are an adjunct to a whole range of ongoing scientific genetic enquiry using innovative new technologies and bioinformatics to search out the genetics and life-style patterns of longevity. Personal insights from nonagenarians themselves provide important information about beliefs, behaviours and social circumstances which further enrich our understanding of the ageing-well phenotype (Baltes and Baltes 1990).

We need to ask ourselves if the psychological characteristics which nonagenarians demonstrate are primarily genetic or cultural—or if the two are inextricably linked through behavioural genetics? Do our behaviours, our choices, our family and social context influence and imprint our genes, so that each new generation takes forward behaviours and family cultural influences in a way which to date cannot be measured, but only surmised from our understanding of social networks, experiences and trans-generational influences (Laland et al. 2010; Heijmans et al. 2008; Pembrey et al. 2014).

The insights related by nonagenarians about how they understand their own good quality ageing, resonate with present-day public health campaigns encouraging people to adopt better life-styles and behaviours in order to set a better life course (Thaler and Sunstein 2008; Marteau et al. 2011; Department of Health 2010). They are living examples of how a combination of factors—family genes, behaviours and beliefs and perhaps a healthy dose of good luck—seem to have improved their chance of living longer and with a better quality of life.

The message for everyone is that taking better care of ourselves, our social networks and actively making life-style choices can increase the chance of ‘*ageing long and ageing well’* and improve the longevity dividend for each person and for society (Olansky et al. 2007).
